# Supplementary material for: Cinnamomum cassia Alleviates Neuropsychiatric Lupus in a Murine Experimental Model
Source: Nutrients. 2025 May 27;17(11):1820. doi: 10.3390/nu17111820 (PMC12157319; doi:10.3390/nu17111820)
Supplement: Supplementary file 1 [file nutrients-17-01820-s001.zip › nutrients-3604948-supplementary.pdf]

Methods:

1. Molecular docking

The 3D structure of TLR7 coupled to imiquimod was first extracted from "Protein Data Bank" with high resolution (<5A°). The 3D structure of cinnamaldehyde, the main active component of cinnamon, was then extracted from PubChem. Molecular docking was performed to predict the possible interaction of cinnamaldehyde with TLR7, using CB-Dock2.

CB-Dock2 uses AutoDock Vina to perform molecular docking after automatically determining protein binding sites, calculating the center and size of the protein, and customizing the docking box size based on the query ligands. Instead of using a protein's whole surface, blind docking was applied only to the predicted locations. Therefore, the initial stage was cavity detection—the search for putative binding sites. Several top cavities are chosen for further investigation (cavity sorting) based on their size since ligand binding sites are often larger cavities. After that, the docking center is determined and the docking box size is adapted. For molecular docking using AutoDock Vina, these parameters (Center and Size) are required. The linked poses were reranked after the docking process was complete using the docking score (Dock and Rerank). The first conformation was regarded as the optimum binding pose, and the corresponding site for the query ligand was deemed to be the best binding site [1,2].

Finally, BIOVIA Discovery Studio software (Dassault Systèmes, Vélizy-Villacoublay, France) was used for the validation of the docking method and detailed visualization of the binding sites with the respective chemical interaction types.

2. Hippocampal cell culture and cellular thermal stability assay (CETSA)

Hippocampal cells were obtained as previously described [3,4]. In brief, brains were harvested after the animals were adequately anaesthetized, and then the olfactory lobes and the cerebellum were resected, and a sagittal section was done at the hippocampus level. The tissue was cut into small pieces and digested with type V collagenase. Cells were cultured in Dulbecco's Modified Eagle's Medium (DMEM), which included 10% fetal bovine serum, 4 mM L-glutamine, 1 mM sodium pyruvate, and 1% penicillin/streptomycin. After five days of culture, hippocampal cells were exposed to imiquimod (10 $\mu g.ml^{-1}$) or cinnamaldehyde (5 μM) for 24 hours. All reagents were from Sigma-Aldrich, Saint Louis, MO, USA.

CETSA was performed as described previously [5–7]. Neuronal cells were exposed to imiquimod, cinnamaldehyde or vehicle control (DMSO) for 24 hours. The cells were then collected, suspended in PBS and dispatched into different tubes. Each aliquot was subjected to thermal denaturation by heating at a series of defined temperatures (50°C, 60°C, 70°C and 80°C) for 10 minutes, followed by rapid cooling on ice. Cells were then solubilized using a non-denaturing lysis buffer containing protease and phosphatase inhibitors. The lysates were spun down to remove the precipitated proteins and the supernatants were collected to assess the thermal stability of the target protein using a dot blot assay (Cleaver Scientific, Warwickshire, UK) with a TLR7 antibody (ab24184; Abcam, Cambridge, UK). After incubation with the secondary antibody, protein signals were quantified using Image Studio Lite Ver 5.2 (LI-COR, NE, USA) and normalized against the unheated control to assess thermal stability in the different conditions.

Results:

Cinnamaldehyde interacts with TLR7

Molecular docking study reveals that cinnamaldehyde, a principal constituent of cinnamon, interacts with TLR7 in multiple sites and may impede TLR7 activation by imiquimod. Imiquimod binds to site 1 in the TLR7 dimerization interface and activates TLR7 dimer. Imiquimod interacts with TLR7 via three hydrogen bonds at cysteine 189 (CYS189), tyrosine 190 (TYR190), and serine 192 (SER192), along with a covalent bond at asparagine 215 (ASN215). When the TLR7/Imiquimod couple was docked, with cinnamaldehyde, the latter seemed to lodge in the same pocket of the TLR7 homodimer interacting with ASN215 by a hydrogen bond. Three other non-covalent interactions are also present at proline 261 (PRO261), CYS270, and TYR468.

Cellular thermal shift assay was performed to validate the molecular docking predictions on the interaction of cinnamon with TLR7. Cinnamon demonstrated a modulating effect on TLR7 stability in neuronal cells (Supplementary figure). CETSA results revealed that TLR7 exhibited enhanced thermal stability in the presence of cinnamaldehyde compared to cells treated with vehicle alone, as indicated by a higher protein expression under heat conditions. Imiquimod was used as a positive control and enhanced TLR7 thermal stability as expected (Figure 5).

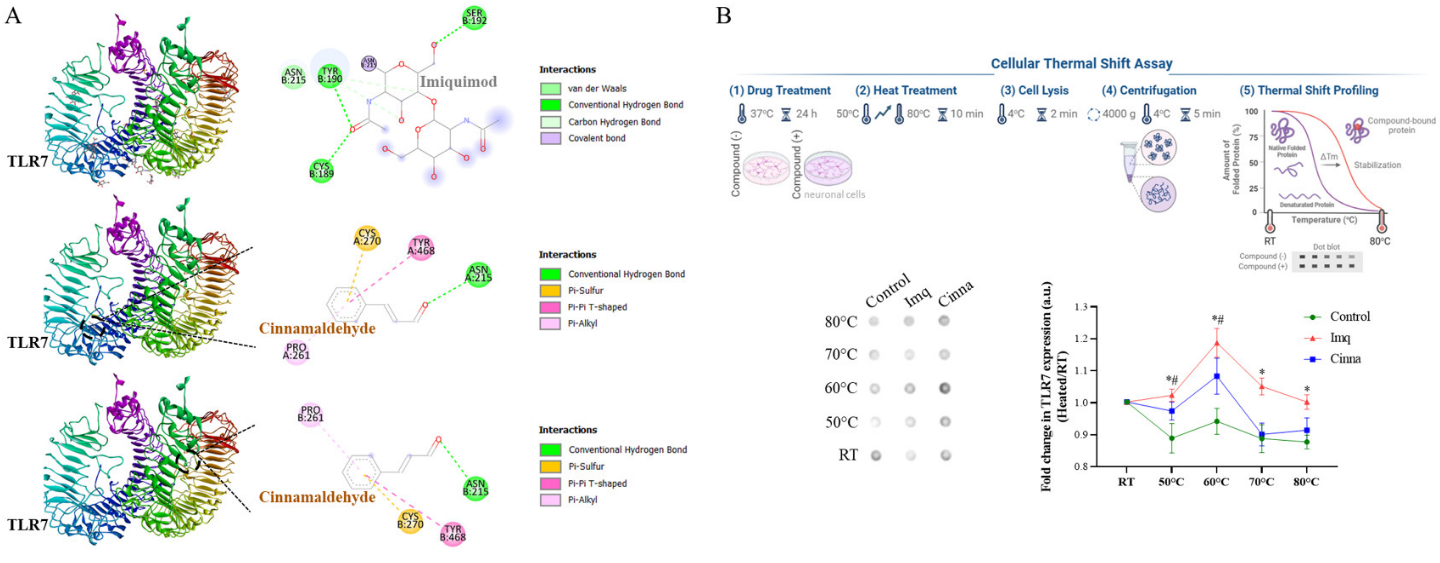

Supplemental Figure 1

References

1. Liu, Y.; Yang, X.; Gan, J.; Chen, S.; Xiao, Z.-X.; Cao, Y. CB-Dock2: Improved Protein-Ligand Blind Docking by Integrating Cavity Detection, Docking and Homologous Template Fitting. *Nucleic Acids Res* **2022**, *50*, W159–W164, doi:10.1093/nar/gkac394.
2. Yang, X.; Liu, Y.; Gan, J.; Xiao, Z.-X.; Cao, Y. FitDock: Protein-Ligand Docking by Template Fitting. *Brief Bioinform* **2022**, *23*, bbac087, doi:10.1093/bib/bbac087.
3. Moutin, E.; Hemonnot, A.-L.; Seube, V.; Linck, N.; Rassendren, F.; Perroy, J.; Compan, V. Procedures for Culturing and Genetically Manipulating Murine Hippocampal Postnatal Neurons. *Front Synaptic Neurosci* **2020**, *12*, 19, doi:10.3389/fnsyn.2020.00019.
4. Kaech, S.; Banker, G. Culturing Hippocampal Neurons. *Nat Protoc* **2006**, *1*, 2406–2415, doi:10.1038/nprot.2006.356.
5. Jafari, R.; Almqvist, H.; Axelsson, H.; Ignatushchenko, M.; Lundbäck, T.; Nordlund, P.; Martinez Molina, D. The Cellular Thermal Shift Assay for Evaluating Drug Target Interactions in Cells. *Nat Protoc* **2014**, *9*, 2100–2122, doi:10.1038/nprot.2014.138.
6. Dayalan Naidu, S.; Dikovskaya, D.; Moore, T.W.; Dinkova-Kostova, A.T. Detection of Thermal Shift in Cellular Keap1 by Protein-Protein Interaction Inhibitors Using Immunoblot- and Fluorescence Microplate-Based Assays. *STAR Protoc* **2022**, *3*, 101265, doi:10.1016/j.xpro.2022.101265.
7. T, I.; T, O.; M, I.-Y.; M, M.; S, U.; M, K.; M, Y.; S, S.; M, N.; T, Y.; et al. CETSA Quantitatively Verifies in Vivo Target Engagement of Novel RIPK1 Inhibitors in Various Biospecimens. *Scientific reports* **2017**, *7*, doi:10.1038/s41598-017-12513-1.
